# Supplementary material for: Neurogenesis-dependent transformation of hippocampal memory traces during systems consolidation
Source: bioRxiv. 2025 Jun 6:2025.06.05.658072. Preprint. [Version 1] doi: 10.1101/2025.06.05.658072 (PMC12157470; doi:10.1101/2025.06.05.658072)
Supplement: Supplement 1 [file NIHPP2025.06.05.658072v1-supplement-1.pdf]

bioRxiv preprint doi: https://doi.org/10.1101/2025.06.05.658072; this version posted June 6, 2025. The copyright holder for this preprint (which was not certified by peer review) is the author/funder, who has granted bioRxiv a license to display the preprint in perpetuity. It is made available under aCC-BY 4.0 International license.

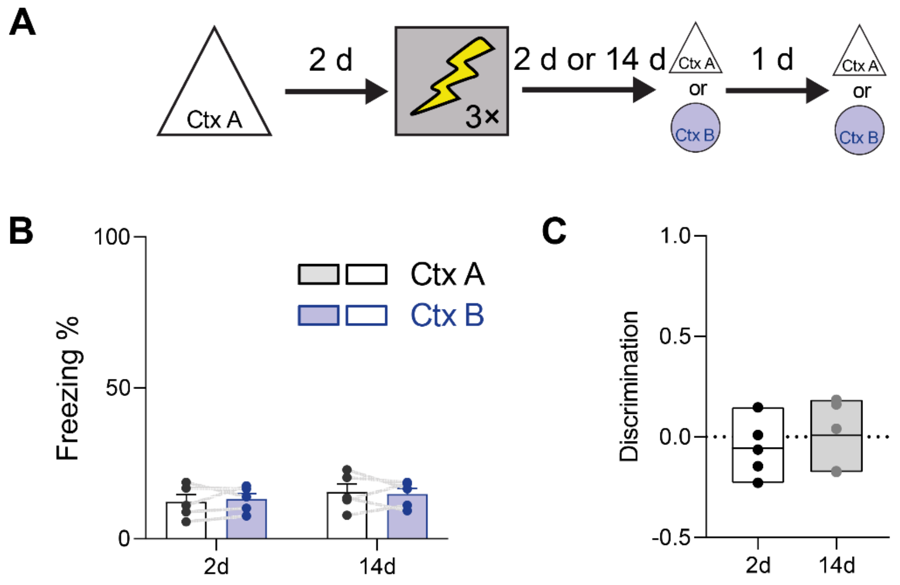

Figure S1. In absence of photostimulation mice do not generalize to contexts A or B.

(A) Mice were pre-exposed to context A., and 2 d later trained in context C (in absence of photostimulation). They were then tested in either contexts A or B either 2 or 14 d post-conditioning. (B) Mice did not freeze in either contexts A or B (2-way ANOVA, main context effect: $F_{1,8}$ = 0.00, P = 0.95; main delay effect: $F_{1,8}$ = 0.74, P = 0.42; stimulation × delay interaction: $F_{1,8}$ = 0.32, P = 0.59). (C) No change in the discrimination between A and B at either of the delays was observed (unpaired t-test, t_8 = 0.64 P = 0.54).

bioRxiv preprint doi: https://doi.org/10.1101/2025.06.05.658072; this version posted June 6, 2025. The copyright holder for this preprint (which was not certified by peer review) is the author/funder, who has granted bioRxiv a license to display the preprint in perpetuity. It is made available under aCC-BY 4.0 International license.

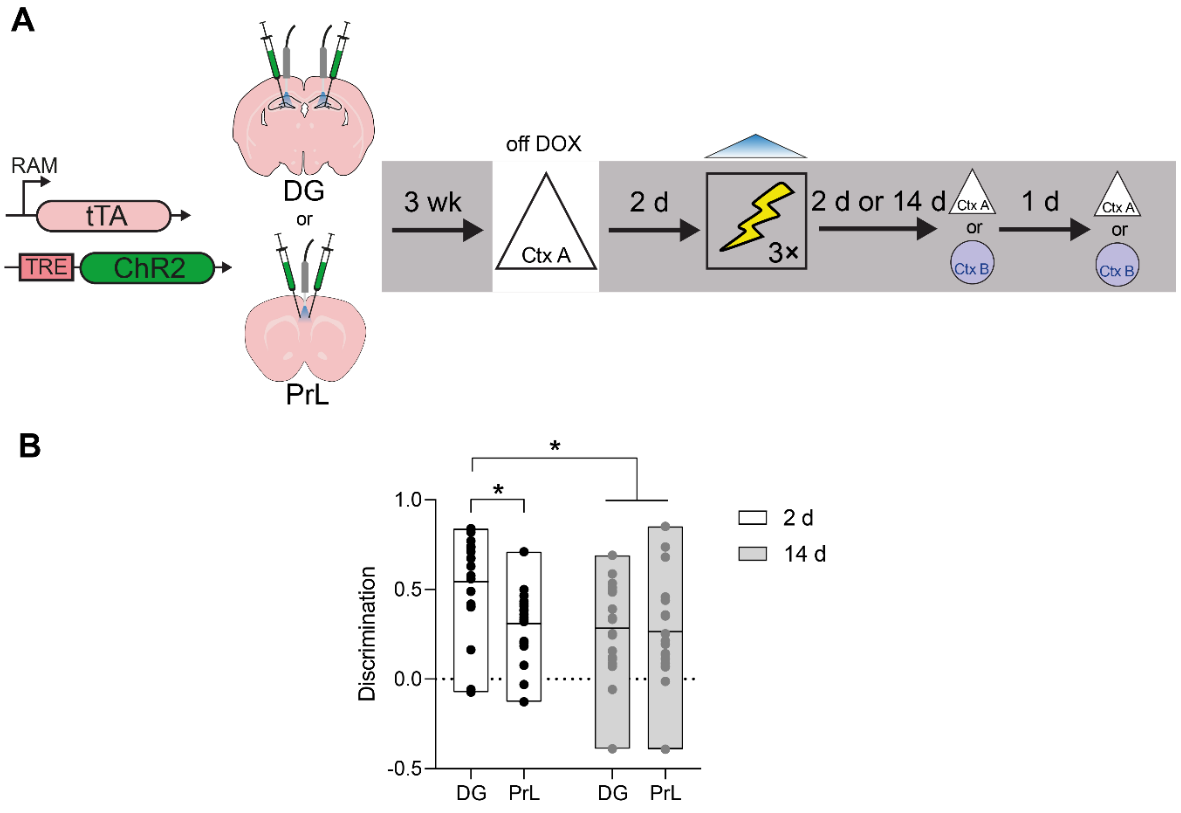

Figure S2. Direct comparison of DG and PrL engram resolution at recent and remote delays.

(A) Experimental design (same as Fig. 2 and Fig. 3). (B) Discrimination declines with delay for DG, but not PrL, engrams (2-way ANOVA, main region effect: $F_{1,73}$ = 5.72, P = 0.02; main delay effect: $F_{1,73}$ = 5.28, P = 0.02; region × delay interaction: $F_{1,73}$ = 4.19, P = 0.04). ∗ $P \le 0.05$, ∗∗ $P \le 0.01$, ∗∗∗ $P \le 0.001$, ∗∗∗∗ $P \le 0.0001$.
